# The Role of the Mesopancreas in Pancreatic Neuroendocrine Neoplasms

**DOI:** 10.3390/jcm15093270

**Published:** 2026-04-24

**Authors:** Stephan O. David, Ahmad. B. Sultani, Andrea Alexander, Sascha Vaghiri, Irene Esposito, Wolfram T. Knoefel, Sami A. Safi

**Affiliations:** 1 Department of Surgery (A), Heinrich-Heine-University and University Hospital Duesseldorf, Moorenstr. 5, 40225 Duesseldorf, Germany; stephanoliver.david@med.uni-duesseldorf.de (S.O.D.); ahmadbaktash.sultani@med.uni-duesseldorf.de (A.B.S.); andrea.alexander@med.uni-duesseldorf.de (A.A.); sascha.vaghiri@med.uni-duesseldorf.de (S.V.); 2 Institute of Pathology, Heinrich-Heine-University and University Hospital Duesseldorf, Moorenstr. 5, 40225 Duesseldorf, Germany; irene.esposito@med.uni-duesseldorf.de

**Keywords:** neuroendocrine neoplasms, NET, NEC, mesopancreas, mesopancreatic excision

## Abstract

**Background**: Pancreatic neuroendocrine neoplasms (PanNENs) represent a heterogeneous tumor entity with a steadily rising incidence, mainly due to advances in imaging and growing diagnostic awareness. In pancreatic ductal adenocarcinoma (PDAC), the mesopancreas (MP) has been identified as a frequent site of microscopic tumor spread and a key determinant of circumferential resection margin (CRM) status, leading to the concept of standardized mesopancreatic excision (MPE). While its oncological relevance in PDAC is increasingly recognized, the role of the mesopancreas in PanNENs remains unclear. This study aimed to systematically evaluate mesopancreatic infiltration in PanNENs and to identify associated clinicopathological predictors. **Methods**: Consecutive patients undergoing oncological pancreatoduodenectomy, spleen-preserving distal pancreatectomy, or distal splenopancreatectomy for PanNENs and PanNECs were included. The mesopancreas was histopathologically examined for tumor infiltration within CRM assessment. **Results**: MP infiltration was detected in 60% of patients. It was associated with higher Ki-67 index, larger tumor size, lymph node involvement, venous invasion, and positive CRM status. A Ki-67 index ≥ 5% and tumor size ≥ 21.5 mm were identified as predictors of MP infiltration. Higher T stage predicted reduced overall survival (OS), whereas MP infiltration, lymphatic (L1) and venous (V1) invasion, and Ki-67 ≥ 5% were associated with impaired disease-free survival (DFS). **Conclusions**: Mesopancreatic infiltration is frequently present in PanNENs and correlates with aggressive tumor characteristics. Given its association with CRM positivity and reduced DFS, consideration of the mesopancreas in staging and surgical strategies appears oncologically justified. Larger studies are required to validate these findings.

## 1. Introduction

Neuroendocrine neoplasms (NENs) constitute a clinically and biologically heterogeneous group of tumors arising from neuroendocrine cells distributed throughout the body [1]. Among these, gastroenteropancreatic neuroendocrine neoplasms (GEP-NENs) represent the most prominent subgroup in terms of incidence and clinical relevance [2,3]. Within this spectrum, pancreatic neuroendocrine neoplasms (PanNENs) have emerged as a particularly diverse and increasingly recognized entity [4,5]. They are broadly categorized into functioning (F-PanNENs) and non-functioning (NF-PanNENs), which typically present later due to a lack of hormone-related symptoms [6,7,8].

PanNENs are further stratified by histopathological criteria, notably the Ki-67 proliferation index and mitotic count, into low-grade (G1), intermediate-grade (G2), and high-grade (G3) tumors. Poorly differentiated G3 neoplasms are classified as neuroendocrine carcinomas (NECs) and are associated with an aggressive clinical course and markedly reduced prognosis [9,10].

The incidence of PanNENs, long underestimated, has increased dramatically in recent decades, largely attributable to advances in imaging modalities and diagnostic awareness [3]. This trend underscores the growing clinical importance of this tumor entity and the pressing need for continued research into its pathophysiology, diagnosis, and treatment. Surgical resection remains the cornerstone of curative treatment, with the primary objective—similar to that in pancreatic ductal adenocarcinoma (PDAC)—being complete (R0) tumor resection [11].

**Figure 1:**
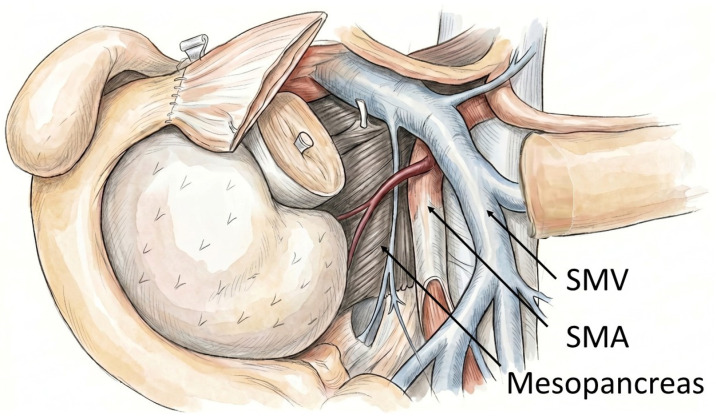
Schematic illustration of the mesopancreas (MP) following pancreatic head dissection. The mesopancreatic region is depicted in relation to the pancreatic head and the major peripancreatic vessels, including the superior mesenteric artery (SMA), superior mesenteric vein (SMV), and the portal vein (PV). The spatial arrangement of the mesopancreas in relation to these vascular structures is shown. Modified from Hirota et al. [22].

**Figure 2:**
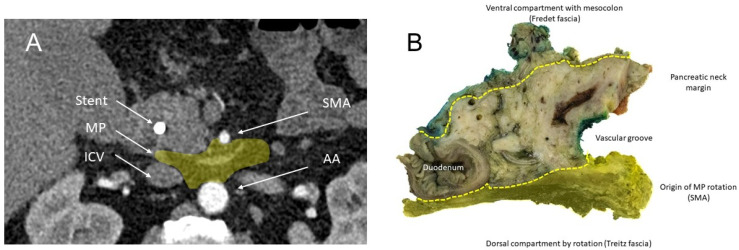
(**A**) Axial contrast-enhanced computed tomography (CT) image in the arterial phase, showing the mesopancreas (MP) in transverse section at the level of the pancreatic head. The mesopancreatic region is located posterior to the pancreatic head and in proximity to the superior mesenteric artery (SMA) and superior mesenteric vein (SMV). The abdominal aorta (AA) and the inferior vena cava (IVC) are visualized dorsally. The image depicts the anatomical relationship between the pancreatic head, SMA, SMV, AA, and IVC. (**B**) Displayed is an axially step-sectioned specimen to orient standardized evaluation. Labeled reference planes include Treitz’s fascia posteriorly and Fredet’s fascia anteriorly. A venous sulcus traces the superior mesenteric vein (SMV). The dorsomedial mesopancreatic pedicle marks the embryologic hinge of mesopancreatic rotation. Mesopancreas is highlighted by yellow hatched area.

The introduction of the circumferential resection margin (CRM) assessment in 2004, following the recommendations of the Royal College of Pathologists, marked a paradigm shift in the histopathological processing of oncological pancreatic resections [12,13]. This protocol, which entails the systematic examination of the ventral, medial, and dorsal specimen surfaces, was further refined by the implementation of the “1 mm rule”—a stringent criterion defining true margin negativity (R0/CRM−) as the absence of tumor cells within 1 mm of any resection margin. As a result, the frequency of genuinely margin-negative resections in pancreatic ductal adenocarcinoma (PDAC) has declined markedly [14,15,16,17]. Particularly the medial and dorsal margins have emerged as critical zones at risk for tumor involvement [14]. Together with the less relevant ventral resection margin, they refer to the mesopancreas (Figure 1 and Figure 2A,B), a peripancreatic area which is embedded by the Treitz and Fredet fascia [18]. In PDACs, the mesopancreas has been shown to be a frequent site of microscopic tumor infiltration, with up to 80% of resected patients demonstrating involvement. Notably, standardized mesopancreatic excision (MPE) has been associated with improved locoregional disease control and reduced recurrence rates [19,20,21].

While the oncological relevance of the mesopancreas in PDAC has been now increasingly established, its role in pancreatic neuroendocrine neoplasms (PanNENs) remains unexplored. To date, no comprehensive studies have investigated the propensity of PanNENs to infiltrate the mesopancreas or assessed the factors that may predispose to such invasion. This study seeks to fill this critical gap by systematically evaluating the infiltration patterns of PanNENs into the mesopancreas and identifying associated clinicopathological parameters. Our findings aim to broaden the current understanding of PanNEN tumor biology and potentially refine surgical strategies for this heterogeneous tumor entity.

## 2. Materials and Methods

Between 2015 and 2025, patients diagnosed with pancreatic neuroendocrine tumors (PanNENs), including NF-PanNEN, F-PanNEN and PanNEC, treated with pancreatoduodenectomy (PD), spleen-preserving distal pancreatectomy and distal splenopancreatectomy at the University Hospital of Duesseldorf were retrospectively included in this analysis. Patients treated with enucleations, parenchyma-sparing procedures or other non-anatomical resections which do not include the mesopancreatic compartment and therefore preclude assessment of mesopancreatic involvement were excluded. Cases were included regardless of pathological tumor stage or final resection margin status. Neuroendocrine tumors of non-pancreatic origin were excluded. Data were derived from a prospectively collected institutional database. Selection criteria required histologically confirmed PanNENs resected with curative intent and availability of circumferential resection margin (CRM) assessment based on a standardized histopathological protocol.

All tumors were staged according to the 8th edition of the UICC TNM classification system. All histopathological data were taken from initial pathological reporting. Since the dorsal part of the mesopancreas is the most limited area for R0 resection, analysis of the mesopancreas was focused on this area (Figure 2B). Mesopancreatic (MP) infiltration was defined as the histopathological presence of tumor cell infiltration within the mesopancreatic adipose or connective tissue compartment, located between the posterior pancreatic tissue and the fascial Treitz capsule. MP positivity (MP+) was assigned when viable tumor cells extended beyond the pancreatic parenchyma into the mesopancreatic fat. Cases without histological evidence of tumor cells in this compartment were classified as MP-negative (MP−).

Further to this, tumor extensions into the dorsal, medial, and ventral CRM zones were evaluated to ensure precise margin classification. For margin interpretation, the “1 mm rule” was applied consistently. A minimum tumor-free distance of 1 mm or more from the resection margin was classified as R0 with negative CRM involvement (R0[CRM−]). If the margin was less than 1 mm, the case was still considered R0 but defined as CRM-positive (R0[CRM+]).

### 2.1. Operation Procedure

In alignment with the established surgical approach of mesopancreatic excision (MPE) in pancreatoduodenectomy for pancreatic head adenocarcinoma [21,23], the same anatomical and oncological principles were applied to distal pancreatectomy in patients with tumors of the pancreatic body and tail (dPDAC) at our institution [23]. The procedure was however individualized for distal PanNEN patients with or without splenectomy, when preoperative imaging and/or intraoperative findings suggested infiltration of the spleen or splenic vessels.

For PanNENs of the pancreatic head, mesopancreatic excision during structured pancreatoduodenectomy was performed independent of biological behavior. Likewise, for total mesocolic excision for right-sided colonic cancers, the Treitz fascia was utilized during the Kocher maneuver in order to visualize the origin of the supermesenteric artery and vein as well as the celiac trunk during early steps of the procedure.

In PanNENs arising from the pancreatic body and tail, surgical therapy was tailored according to the biological behavior (tumor size and >KI index). In high-risk PanNENs, surgical therapy was translated from PDAC patients. Exposure of the greater sac enabled visualization of the superior mesenteric vein (SMV) and artery (SMA), which were visualized on their ventral and left lateral surfaces for high-risk PanNENs of the pancreatic body and tail. The inferior mesenteric vein (IMV) was identified, if necessary due to the proximity of the tumor, and divided prior to entering the mesopancreatic compartment. In order to achieve tumor clearance on the superior surface of the pancreatic body and tail, the origin of the splenic artery was visualized from the celiac trunk. Transection was than followed from medially towards the Gerota fascia utilizing an avascular plain between the Treitz and Gerota fascia. The splenic vein was then exposed, including its confluence with the SMV when required. Pancreatic transection was performed with the resection line tailored to tumor localization in order to preserve pancreatic parenchyma whenever possible; transection above the mesenterico-portal axis was therefore not mandatory but applied selectively. If tumor infiltration of the vasculature was suspected, venous resection and reconstruction were carried out as required.

The gastrosplenic ligament was divided close to the greater curvature of the stomach. Dissection proceeded along the celiac trunk and SMA to their origins at the aorta in a controlled and individualized manner. To minimize sympathetic denervation, the left circumference of the SMA was dissected only 180°. In cases of intraoperative suspicion of tumor involvement, the dissection was expanded circumferentially. A paraaortic lymphadenectomy was subsequently performed, proceeding from medial to lateral. Dissection was continued cranially until complete mobilization of the spleen was achieved, and extended to adjacent retroperitoneal structures only when oncologically indicated.

In selected cases without evidence of perisplenic nodal involvement, a spleen-preserving distal pancreatectomy was performed. Here, the splenic artery and vein were preserved, and pancreatic tissue was dissected carefully from the splenic hilum, while adhering to the defined mesopancreatic planes.

In all patients, the aim was a complete mesopancreatic excision with removal of perineural and lymphovascular tissue surrounding the celiac trunk, common hepatic artery, SMA, portal vein/SMV, splenic artery and vein, and IMV, as part of a systematic en bloc resection. The exact sequence and extent of surgical steps were adapted according to tumor location, size, and intraoperative anatomy.

### 2.2. Statistics

Statistical analysis was performed to evaluate both associations and predictive values of clinico-pathological parameters. Non-parametric methods were applied throughout. Continuous or ordinal variables were compared between independent groups using the Mann–Whitney U test. Categorical variables were analyzed using the chi-square test or Fisher’s exact test, as appropriate. Receiver operating characteristic (ROC) curve analysis was conducted to assess the diagnostic or prognostic performance of continuous variables, and the area under the curve (AUC) was calculated to quantify discriminative ability. To assess overall survival (OS) and disease-free survival (DFS), Kaplan–Meier survival curves were generated. Differences between groups were evaluated using the log-rank test. Univariate analyses were performed for both oncological outcome parameters. For DFS, a multivariate analysis using the Cox proportional hazards regression model was additionally conducted in cases with multiple significant prognostic factors. Variables including N-stage, Ki-67 index, lymphatic invasion (L), and venous invasion (V) were entered into the model based on their clinical relevance and significance in univariate analysis, representing established prognostic factors in PanNENs. A forward stepwise likelihood ratio approach was applied, while limiting the number of variables due to the small number of events. Under these conditions, formal testing of the proportional hazards assumption is of limited interpretability.

All statistical analyses were carried out using IBM SPSS Statistics for Windows, Version 26.0 (IBM Corp., Armonk, NY, USA). A two-sided *p*-value of <0.05 was considered statistically significant.

This study was conducted in accordance with the ethical standards of the Declaration of Helsinki and the principles of Good Clinical Practice. Approval was obtained from the Institutional Review Board (IRB) of the Medical Faculty, Heinrich Heine University Duesseldorf (IRB-no. 2020–1990, 2019–473, 2019–473-1 and 2019–473-2).

## 3. Results

### 3.1. Demographic Data

A total of 49 patients who underwent pancreatic surgery for pancreatic neuroendocrine neoplasms (PanNENs) during the study period were initially identified, and all corresponding specimens were reviewed histopathologically. Fourteen patients were excluded because enucleations, parenchyma-sparing procedures or other non-anatomical resections do not include the mesopancreatic compartment and therefore preclude assessment of mesopancreatic involvement. The final study cohort thus consisted of 35 patients who underwent anatomical pancreatic resection with curative intent and for whom complete circumferential resection margin (CRM) analysis, including evaluation of the mesopancreas, was available. Demographic data are summarized in Table 1. The cohort comprised 23 (65.7%) patients with non-functioning PanNENs (NF-PanNENs), nine (25.8%) with functioning PanNENs (F-PanNENs), and three (8.6%) patients with pancreatic neuroendocrine carcinoma (PanNEC). Among the hormonally active tumors, eight patients had insulinomas and one patient had a gastrinoma; two insulinomas occurred in patients with multiple endocrine neoplasia type 1 (MEN1). Concerning grading status, eleven (31.4%) patients were classified as G1, 21 (60.0%) as G2 and three (8.6%) as G3. The median age at surgery was 58 years (21–81). Among the 35 patients included in this study, pancreatoduodenectomy was the most frequently performed surgical procedure (37.1%), followed by spleen-preserving distal pancreatectomy (34.3%) and distal pancreatectomy with splenectomy (28.6%). CRM positivity was observed in nine patients at the dorsal margin and in nine patients at the medial margin. Notably, there was an overlap of two patients who demonstrated positivity at both margins. None of the patients had received an R1 resection. Notably, mesopancreatic infiltration was observed in 21 (60.0%) cases classified as MP-positive, while 14 patients (40.0%) showed no evidence of mesopancreatic involvement.

### 3.2. Correlation Analysis According to Infiltration Status in the Mesopancreas (MP)

**Figure 3:**
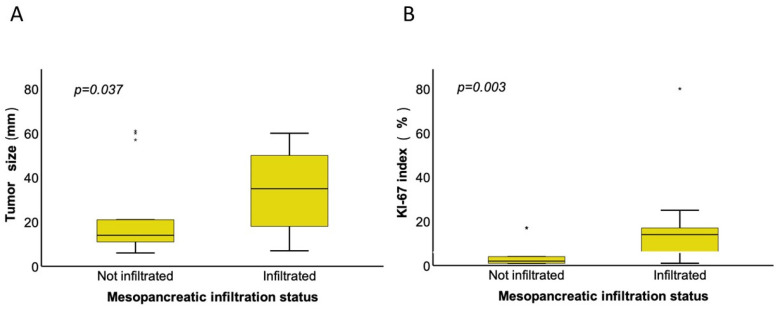
(**A**) Boxplots illustrating the correlation of tumor size with mesopancreatic infiltration status in PanNENs. Larger tumor size (in mm) was significantly associated with a positive mesopancreatic infiltration status (Mann–Whitney U test, *p* < *0.05*). (**B**) Boxplot presenting correlation of Ki-67 index with mesopancreatic infiltration status in PanNENs. Higher Ki-67 indices were significantly associated with a positive mesopancreatic infiltration status (Mann–Whitney U test, *p* < *0.05*). The asterisks indicate levels of statistical significance. Specifically, * denotes *p* < *0.05* and ** denotes *p* < *0.001*.

In the analyzed cohort, MP infiltration was significantly associated with more advanced tumor characteristics (Table 2). Patients with MP infiltration had markedly higher rates of lymph node involvement (71.4% vs. 14.3%; OR = 15.00; 95% CI: 2.55–88.17; *p < 0.001*), venous invasion (33.3% vs. 0%; OR = 15.00; 95% CI: 0.79–283.60; *p = 0.016*), and higher-grade tumors (*p = 0.007*) compared with MP-negative patients. Furthermore, positive circumferential resection margins (CRMs) at both dorsal and medial sites were strongly linked to MP infiltration (dorsal R0CRM+: 42.8% vs. 0%; OR = 22.04; 95% CI: 1.17–414.60, *p = 0.004*; medial R0CRM+: 38.1% vs. 7.1%; OR 8.00; 95% CI: 0.87–73.58; *p = 0.040*) (Table 2). We further subgrouped patients according to the tumor location within the pancreas (Supplemental Tables S1 and S2). Histopathological staging variables were homogenously distributed between pancreatic head and distal pancreatic PanNENs (Supplemental Table S1). Patients undergoing distal splenopancreatectomy showed significantly higher rates of nodal involvement (*p = 0.011*), perineural invasion (*p = 0.041*), lymphatic invasion (*p = 0.015*), and vascular invasion (*p = 0.015*) compared with patients undergoing spleen-preserving distal pancreatectomy, whereas mesopancreatic infiltration (*p = 0.639*) did not differ significantly between the two groups (Supplemental Table S2). Tumor size was greater in patients with MP infiltration (*p = 0.037*) (Figure 3A). The Ki-67 index was elevated in the MP-positive group (median 14% vs. 2%; *p = 0.003*) (Figure 3B).

### 3.3. Predictive Value of Ki-67 Index and Tumor Size for Mesopancreatic Infiltration

**Figure 4:**
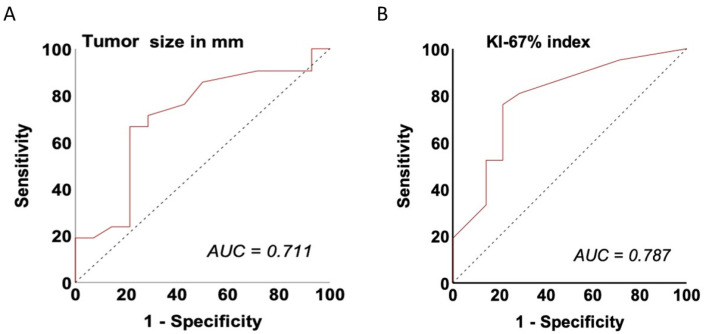
(**A**) Receiver operating characteristic (ROC) curve with corresponding area under the curve (AUC = 0.711; sensitivity 0.667; specificity 0.786; 95% CI: 0.552–0.900) for tumor size (≥21.5 mm) in predicting mesopancreatic infiltration in patients with PanNENs. (**B**) Receiver operating characteristic (ROC) curve with corresponding area under the curve (AUC = 0.787; sensitivity 0.762; specificity 0.786; 95% CI: 0.609–0.939) for a Ki-67 index ≥ 5% in predicting mesopancreatic infiltration in patients with PanNENs.

Logistic regression confirmed a Ki-67 index ≥ 5% as a predictor, corresponding to an 11.73-fold higher likelihood of mesopancreatic involvement (OR = 11.73; 95% CI: 2.31–59.54; *p* = 0.003) (Supplemental Table S3). In an additional exploratory combined logistic regression model including both tumor size and Ki-67 index, only Ki-67 ≥ 5% remained significantly associated with mesopancreatic infiltration (OR = 9.51; 95% CI: 1.68–53.72; *p* = 0.011). The predictive model achieved good discriminatory performance, with an AUC of 0.787 (sensitivity 0.762; specificity 0.786; 95% CI: 0.609–0.939) (Figure 4B).

**Figure 5:**
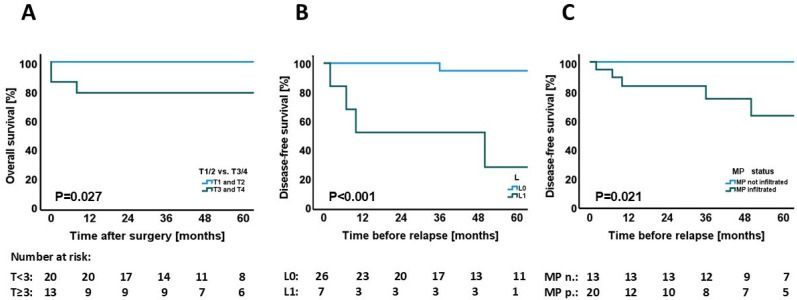
Kaplan–Meier survival analyses of patients with pancreatic neuroendocrine tumors. (**A**) Overall survival (OS) stratified by tumor stage (T1/2 vs. T3/4) demonstrated a significantly reduced OS in patients with advanced tumors (*p = 0.027*). (**B**) Disease-free survival (DFS) according to lymphovascular invasion (L0 vs. L1) revealed markedly impaired DFS in patients with L1 tumors (*p < 0.001*). (**C**) DFS stratified by mesopancreatic (MP) infiltration status showed a significantly decreased DFS in MP-positive compared to MP-negative cases (*p = 0.021*).

For tumor size, ROC analysis identified a cut-off of ≥ 21.5 mm, with MP infiltration occurring more frequently above this threshold (*p = 0.001*; Supplemental Table S4). Logistic regression analysis demonstrated that tumors ≥ 21.5 mm were associated with MP infiltration, carrying a 7.33-fold increased probability of involvement (OR = 7.33; 95% CI: 1.53–35.11; *p = 0.013*). The discriminatory ability of this size-based model was acceptable, with an AUC of 0.711 (sensitivity 0.667; specificity 0.786; 95% CI: 0.552–0.900) (Figure 5A).

### 3.4. Association Between Individual Clinical and Histopathological Parameters and Overall Survival

Follow-up data of all 35 patients was obtained using official records from the registration office. Two patients deceased in the first 30-day postoperative period and were excluded for further analysis (mortality rate = 5.71%). The median OS of the remaining 33 patients was 49 months. Univariate analysis of all clinicopathological parameters (Supplemental Table S5) identified only advanced T-stage (T1/2 vs. T3/4) as significantly associated with overall survival (*p = 0.027*) (Figure 5A). Other factors, including nodal involvement (*p = 0.059*), Ki-67 ≥ 5% (*p = 0.094*), and grading (*p = 0.322*), failed to reach statistical significance in this analysis, despite trends consistent with the above findings.

### 3.5. Association Between Individual Clinical and Histopathological Parameters and Disease-Free Survival

Follow-up data of relapse-free survival was available in all 33 PanNEN patients (median follow-up period was 49 months); the median RFS was 41 months. Univariate disease-free survival (DFS) analysis (Table 3) revealed significant prognostic implications for nodal status (*p = 0.035*), Ki-67 ≥ 5% (*p = 0.021*), lymphatic invasion (*p < 0.001*) (Figure 5B), venous invasion (*p < 0.001*), and notably MP infiltration *(p = 0.021*) (Figure 5C). Notably, a total of five recurrences were observed (15.2%), with three occurring in the liver (after 7, 10, and 50 months), and one at the stomach (after 2 months). One locoregional recurrence was detected after 36 months.

Multivariate Cox regression analysis identified lymphatic invasion as the sole independent predictor of reduced DFS (hazard ratio [HR]: 22.34, 95% CI: 2.49–200.57, *p = 0.006*) (Table 3).

## 4. Discussion

Over the past decades, the reported incidence of pancreatic neuroendocrine neoplasms (PanNENs) has risen substantially. This trend is largely attributable to advances in cross-sectional imaging and improved diagnostic awareness, which have led to the detection of an increasing number of asymptomatic and early-stage lesions [3,24,25]. As a result, PanNENs have gained clinical relevance, and the need for more precise, biology-driven surgical concepts has become evident. Surgical resection with margin negativity remains the cornerstone of curative treatment in PanNENs, despite recent developments in systemic therapy [26]. Complete tumor clearance (R0) has been shown to be a decisive prognostic factor, similar to other carcinomas of the pancreas [26,27].

The mesopancreas (MP), as an embryologically defined compartment bordered by the Treitz and Fredet fascia, is a key site for tumor invasion in PDAC patients. MP involvement is reported in up to 80% of resected specimens [19,21] and standardized mesopancreatic excision (MPE) improves R0 rates and the average number of lymph nodes harvested and reduces local recurrence [19,23,28,29,30]. Moreover, the meta-analysis by Kim et al. [20] confirmed the favorable safety profile of mesopancreatic excision, revealing no significant differences compared with standard pancreatoduodenectomy in terms of hospital stay, postoperative complications, mortality, reoperation rates, or operative time, thereby supporting its oncological application in appropriately selected patients and experienced centers.

In this context, the growing relevance of preoperative imaging has increasingly been recognized for the evaluation of the mesopancreatic region and surgical planning. We and others were able to demonstrate that preoperative MDCT, particularly mesopancreatic fat stranding [31,32,33] and increased mesopancreatic density [34], correlate with histopathologically confirmed mesopancreatic infiltration in PDAC, supporting the concept of imaging-based risk stratification of the mesopancreas. However, while these findings have been supported by our data and other studies, they have not been consistently confirmed across the literature [35]. Importantly, at our institution, MPE is performed routinely and independent of preoperative imaging findings, and is currently not guided by imaging-based selection criteria.

Although mesopancreatic excision (MPE) is increasingly recognized as an oncologically relevant concept, the lack of a universally accepted operative standard remains a critical issue. Different strategies have been proposed, reflecting both technical and oncological considerations. The no-touch technique, as described by Hirota et al. [22], aims to avoid tumor manipulation by performing vascular dissection prior to tumor mobilization, incorporating pre-aortic tunneling and a reversed Kocher maneuver to achieve early vascular control and mesopancreatic isolation. In contrast, artery-first approaches, as described by Weitz et al. [36], involve identification of the superior mesenteric artery below the pancreas on the left side of the mesentery with subsequent dissection carried upwards, facilitating controlled dissection of the medial mesopancreatic margin. A variation of this concept is the supracolic anterior artery-first approach proposed by Inoue et al. [37], which enables early arterial control via an anterior supracolic route. Despite these procedural differences, all approaches aim to improve oncological radicality through optimized control of the mesopancreatic plane.

However, the MP infiltration has not been investigated in PanNENs, and robust evidence is lacking as to whether mesopancreatic excision (MPE) is justified in this tumor entity or should even be regarded as an integral part of surgical management.

In our cohort, MP infiltration was observed in 60% of PanNENs, which approximates the infiltration rates described for PDAC [19,21]. This data underscores the anatomical and oncological parallels between high-risk PanNENs and PDACs, despite their different cellular origin. Furthermore, MP involvement was significantly associated with higher CRM-positive rates at both the dorsal (42.8% vs. 0%; *p* = 0.004) and medial margins (38.1% vs. 7.1%; *p* = 0.040). Consistent with these findings, MP infiltration was also linked to impaired disease-free survival in the univariate analysis (*p* = 0.021), underscoring its clinical relevance as a potential marker of adverse tumor behavior. Taken together, these associations identify the MP as a critical determinant of margin positivity and, consequently, local tumor control in PanNEN surgery.

Furthermore a Ki-67 index ≥ 5% was associated with an 11.73-fold increased risk of MP involvement. Previous studies have demonstrated that a higher Ki-67 index correlates with lymphovascular invasion, perineural spread, and nodal metastasis in neuroendocrine tumors [38,39]. Our results extend these findings by linking proliferative activity to invasion of a defined anatomical compartment. In an additional exploratory combined logistic regression model including both tumor size and Ki-67 index, only Ki-67 ≥ 5% remained significantly associated with mesopancreatic infiltration, whereas tumor size did not retain statistical significance. Given that Ki-67 can be obtained preoperatively via biopsy or fine-needle aspiration, it may serve as a clinically relevant marker to guide risk stratification and support decision-making regarding the indication for mesopancreatic excision in higher-risk patients. Moreover, the findings of Chopde et al. support the idea that a Ki-67 index exceeding 5% is associated with an increased rate of local recurrence following curative resection [40]. In line with these observations, our data further demonstrate that a Ki-67 index ≥ 5% correlates with significantly reduced disease-free survival, underscoring its prognostic relevance in PanNENs. Moreover, we demonstrated that a tumor size ≥ 21.5 mm was associated with a 7.33-fold higher risk of mesopancreatic involvement, which, as previously mentioned, constitutes a risk factor for incomplete resection. Unsurprisingly, larger tumor size was also significantly associated with reduced overall survival. These findings are consistent with previous reports, which likewise identified tumor size as an independent prognostic factor for overall survival in patients with pancreatic neuroendocrine tumors [41,42,43].

Limitations include the retrospective single-center design, the wide study period, and the limited sample size. However, all surgical procedures and histopathological evaluations were performed by the same team, thereby reducing inter-operator and inter-observer variability. Nevertheless, the small number of patients inevitably limits statistical power, and results from multivariable analyses, particularly regarding Ki-67 index and tumor size, must be interpreted with caution. The limited number of events, particularly in the disease-free survival analysis, restricts the robustness of the multivariable Cox regression model and may result in imprecise effect estimates, as reflected by the wide confidence intervals. This is particularly evident for lymphatic invasion, which showed a high hazard ratio with a wide confidence interval, indicating limited estimate stability; therefore, the findings of the multivariable analysis should be interpreted as exploratory rather than definitive. Furthermore, the derivation of cut-offs from ROC analysis and their subsequent evaluation within the same cohort may have led to an overestimation of effect sizes and should therefore be interpreted with caution. Importantly, these findings are in line with the current literature reporting Ki-67 and tumor size as key prognostic and biological determinants in PanNENs [43]. Larger, adequately powered studies are needed to provide more robust evidence and to better elucidate the relative contribution of different prognostic factors in PanNENs. Furthermore, the analysis was restricted to patients undergoing anatomical pancreatic resections, which may have introduced a selection bias towards larger and biologically more aggressive tumors, as smaller PanNENs managed by enucleation were not included. These cases could not be evaluated for mesopancreatic involvement, since the mesopancreas is not resected in parenchyma-sparing procedures. Moreover, the cohort included different types of pancreatic resections: pancreatoduodenectomy, spleen-preserving distal pancreatectomy, and distal splenopancreatectomy—resulting in a relatively diverse set of operative procedures, which further limits the comparability of the results.

Taken together, our results suggest that a mesopancreatic excision may be especially justified in PanNENs exhibiting both an increased Ki-67 index (>5%) and a larger tumor diameter (≥21.5 mm), as these combined features indicate a markedly elevated risk of mesopancreatic involvement. In these higher-risk constellations, current guidelines already advocate anatomical pancreatic resection with lymph node assessment, an approach that is further supported by our findings [44,45,46]. Although MP infiltration did not emerge as an independent prognostic factor in multivariable survival analysis, this finding must be interpreted with caution given the limited cohort size and associated risk of statistical underpowering. PanNENs are very rare tumors of the pancreas which is underlined by our unicentric study cohort. To overcome this, we performed subgroup analysis stratifying the patients according to the tumor location in the pancreas and it revealed a homogenous distribution of staging and tumor-biology variables. Our observations indicate that the distinction between true anatomical tumor extension (MP+ vs. MP−) and underlying aggressive biology is not entirely clear-cut in PanNENs. A higher Ki67 index as well as an increased tumor size significantly correlated with true tumor extension into the mesopancreas. MP positivity closely paralleled several high-risk features, yet its association with increased CRM involvement highlights the anatomical relevance of this compartment. Thus, while a definitive independent effect on outcome cannot be confirmed, it likewise cannot be excluded. This study should primarily serve as a proof-of-concept in order to gather more insights and focus more attention on the mesopancreas in patients diagnosed with PanNENs. This provides sufficient rationale to consider the mesopancreas not only during surgical resection but also in preoperative staging. In summary, these data strongly suggest a considerable oncological relevance of the MP in PanNENs and consecutive MPE.

## 5. Conclusions

In conclusion, MP infiltration is a frequent event in PanNENs with aggressive biology and correlates with higher CRM-positive rates and reduced DFS. These findings provide robust oncological rationale for considering the mesopancreas in PanNENs with high-risk features to achieve optimal local control. Prospective, ideally multicentric studies are warranted to validate these observations and to establish clear patient selection criteria for systematic MPE in the management of PanNENs.

## Figures and Tables

**Table 1:** Demographic data of all 35 patients. Staging is revised to the 8th edition of the UICC TNM classification of malignant tumors.

**Age in Years**		
Median (range)	58 (21–81)	
**Gender**	**n**	**%**
Male	23	65.7
Female	12	34.3
**Neuroendocrine subgroup**		
NF-PanNEN	23	65.7
F-PanNEN	9	25.8
PanNEC	3	8.6
**Tumor size in mm**		
Median (range)	21 (6–200)	
**T-stage**		
T1 and T2	21	60.0
T3 and T4	14	40.0
**N-stage**		
N0	18	51.4
N1	17	48.6
**Ki-67 Index in %**		
Median (range)	6 (1–80)	
**Grading**		
G1	11	31.4
G2	21	60.0
G3	3	8.6
**Pn-status**		
Pn0	28	80.0
Pn1	7	20.0
**L-status**		
L0	28	80.0
L1	7	20.0
**V-status**		
V0	28	80.0
V1	7	20.0
**R-status dorsal**		
R0CRM+	9	25.7
R0CRM-	26	74.3
**R-status medial**		
R0CRM+	9	25.7
R0CRM-	26	74.3
**MP-status**		
MP negative	14	40.0
MP positive	21	60.0
**Type of resection**		
Pancreatoduodenectomy	13	37.1
Spleen-preserving distal pancreatectomy	12	34.3
Distal splenopancreatectomy	10	28.6

CRM = circumferential resection margin; F-PanNEN = functioning pancreatic neuroendocrine neoplasm; G = tumor grading; Ki-67 = Ki-67 proliferation index; L = lymphatic invasion; MP = mesopancreas; N = nodal stage; NF-PanNEN = non-functioning pancreatic neuroendocrine neoplasm; PanNEC = pancreatic neuroendocrine carcinoma; Pn = perineural invasion; T = tumor stage; UICC = Union for International Cancer Control; V = venous invasion.

**Table 2:** Statistical significance was calculated using the chi-square test and Fisher’s exact test, as appropriate.

	MP Infiltration + n = 21		MP Infiltration − n = 14		*p-Value*
**Sex**	**n**	**%**	**n**	**%**	*0.383*
Male	15	71.4	8	57.1	
Female	6	28.6	6	42.9	
**Neuroendocrine subgroup**					*0.072*
NF-PanNEN	15	71.4	8	57.1	
F-PanNEN	3	14.3	6	42.9	
PanNEC	3	14.3	0	0.0	
**T-stage**					*0.067*
T1 and T2	10	47.6	11	78.6	
T3 and T4	11	52.4	3	21.4	
**N-stage**					<0.001 **
N0	6	28.6	12	85.7	
N1	15	71.4	2	14.3	
**Grading**					0.007 **
G1	3	14.3	9	64.3	
G2	15	71.4	5	35.7	
G3	3	14.3	0	0.0	
**Pn-status**					*0.121*
Pn0	15	71.4	13	92.9	
Pn1	6	28.6	1	7.1	
**V-status**					0.016 *
V0	14	66.6	14	100.0	
V1	7	33.3	0	0.0	
**L-Status**					*0.121*
L0	15	71.4	13	92.9	
L1	6	28.6	1	7.1	
**R-status dorsal**					0.004 **
R0CRM+	9	42.8	0	0.0	
R0CRM−	12	57.2	14	100.0	
**R-status medial**					0.040 *
R0CRM+	8	38.1	1	7.1	
R0CRM−	13	61.9	13	92.9	
**Type of resection**					*0.618*
Pancreatoduodenectomy	9	42.8	4	28.6	
Spleen-preserving distal pancreatectomy	6	28.6	6	42.8	
Distal splenopancreatectomy	6	28.6	4	28.6	

CRM = circumferential resection margin; F-PanNEN = functioning pancreatic neuroendocrine neoplasm; G = tumor grading; L = lymphatic invasion; MP = mesopancreas; N = nodal stage; NF-PanNEN = non-functioning pancreatic neuroendocrine neoplasm; PanNEC = pancreatic neuroendocrine carcinoma; Pn = perineural invasion; R = resection margin status; T = tumor stage; V = venous invasion.** indicates a *p*-value ≤ 0.01; * indicates a *p*-value ≤ 0.05.

**Table 3:** Univariate and multivariate analysis of individual clinical and histopathological parameters regarding disease-free survival.

Univariate Analysis				
		***p-Value***		
Median age (< vs. >median)		*0.654*		
Sex (male vs. female)		*0.421*		
Tumor location (head vs. tail)		*0.384*		
T-stage (T1/T2 vs. T3/T4)		*0.280*		
N-stage (N0 vs. N1)		***0.035*** *		
Ki-67% (<5% vs. ≥5%)		*0.021*		
Grading (G1 vs. G2/G3)		*0.157*		
Pn (Pn0 vs. Pn1)		*0.773*		
L (L0 vs. L1)		***<0.001*** **		
V (V0 vs. V1)		***<0.001*** **		
R-status (R0CRM− vs. R0CRM+)		*0.352*		
MP-status (MP+ vs. MP−)		0.021 *		
**Multivariate analysis**				
	***p-value***		**HR**	**95%CI**
L-status (L0 vs. L1)	*0.006*		22.34	2.49–200.57

CI = confidence interval; CRM = circumferential resection margin; G = tumor grading; HR = hazard ratio; Ki-67 = Ki-67 proliferation index; L = lymphatic invasion; MP = mesopancreas; N = nodal stage; Pn = perineural invasion; R = resection status; T = tumor stage; V = venous invasion. ** indicates a *p*-value ≤ 0.01; * indicates a *p*-value ≤ 0.05.

## Data Availability

The datasets used and/or analyzed during the current study are available from the corresponding author on reasonable request.

## Supplementary Materials

The following supporting information can be downloaded at: https://www.mdpi.com/article/10.3390/jcm15093270/s1, Supplemental Table S1: Correlation analysis of the 35 patients stratified according to tumor location. Statistical significance was calculated by chi squared test and Wilcoxon test. Supplemental Table S2: Correlation analysis of the 22 patients stratified according to resection type. Statistical significance was calculated by chi squared test and Wilcoxon test. ** indicates a *p*-value ≤ 0.01; * indicates a *p*-value ≤ 0.05. Supplemental Table S3: Prediction analysis of the mesopancreatic infiltration status by Ki-67 index in PanNEN patients. Optimal cut-off point for Ki-67 index was computed by ROC analysis. Ordinal scaling of Ki-67 index (<cut-off and >cut-off) was tested for distribution against MP infiltration status (chi-square test). Binary logistic regression analysis was used for prediction analysis. Supplemental Table S4: Prediction analysis of the mesopancreatic infiltration status by tumor size in PanNEN patients. Optimal cut-off point for tumor size was computed by ROC analysis. Ordinal scaling of tumor size (<cut-off and >cut-off) was tested for distribution against MP infiltration status (chi-square test). Binary logistic regression analysis was used for prediction analysis. Supplemental Table S5: Univariate analysis of individual clinical and histopathological parameters regarding overall survival. STROBE Adherence: This study was conducted in accordance with the STROBE (Strengthening the Reporting of Observational Studies in Epidemiology) guidelines, and all relevant recommendations were adhered to in the reporting of this manuscript. The completed STROBE checklist is provided in the Supplemental Files.

## Abbreviations

CRM	Circumferential Resection Margin
DFS	Disease-Free Survival
F-PanNEN(s)	Functioning Pancreatic Neuroendocrine Neoplasm(s)
G1/G2/G3	Grading (low, intermediate, high)
GEP-NEN(s)	Gastroenteropancreatic Neuroendocrine Neoplasm(s)
L1	Lymphatic invasion (positive)
MP	Mesopancreas
MPE	Mesopancreatic Excision
NEC(s)	Neuroendocrine Carcinoma(s)
NEN(s)	Neuroendocrine Neoplasm(s)
NF-PanNEN(s)	Non-Functioning Pancreatic Neuroendocrine Neoplasm(s)
OS	Overall Survival
PanNEC	Pancreatic Neuroendocrine Carcinoma
PanNEN(s)	Pancreatic Neuroendocrine Neoplasm(s)
PD	Pancreatoduodenectomy
PDAC	Pancreatic Ductal Adenocarcinoma
ROC	Receiver Operating Characteristic
R0/CRM−	Resection margin > 1 mm (true negative margin)
R0/CRM+	Resection margin < 1 mm (margin positive despite R0)
SMA	Superior Mesenteric Artery
SMV	Superior Mesenteric Vein
UICC	Union for International Cancer Control
