# Supplementary material for: The Role of the Mesopancreas in Pancreatic Neuroendocrine Neoplasms
Source: J Clin Med. 2026 Apr 24;15(9):3270. doi: 10.3390/jcm15093270 (PMC13163267; doi:10.3390/jcm15093270)
Supplement: Supplementary file 1 [file jcm-15-03270-s001.zip › jcm-4246416-supplementary.pdf]

Supplemental Table S1: Correlation analysis of the 35 patients stratified according to tumor location. Statistical significance was calculated using the chi-square test or Fisher's exact test, as appropriate. ** indicates a p-value ≤ 0.01; * indicates a p-value ≤ 0.05.

	**Pancreatic head** n=13		**Pancreatic tail** n=22		***p-value***
**Sex**	**n**	**%**	**n**	**%**	*0.736*
Male	9	69.2	14	63.6	
Female	4	30.8	8	36.4	
**T-stage**					*0.568*
T1 and T2	7	53.8	14	63.6	
T3 and T4	6	46.2	8	36.4	
**N-stage**					*0.060*
N0	4	30.8	14	63.6	
N1	9	69.2	8	36.4	
**Grading**					*0.540*
G1	4	30.8	8	36.4	
G2	7	53.8	13	59.1	
G3	2	15.4	1	4.5	
**Pn-status**					*0.221*
Pn0	9	69.2	19	86.4	
Pn1	4	30.8	3	13.6	
**V-status**					*0.726*
V0	10	76.9	18	81.8	
V1	3	23.1	4	18.2	
**L-Status**					*0.726*
L0	10	76.9	18	81.8	
L1	3	23.1	4	18.2	
**Ki-67-Index**					*0.184*
Ki-67 Index < 5%	6	58.3	10	30.0	
Ki-67 Index ≥ 5 %	7	41.7	12	70.0	
**MP-Status**					*0.392*
MP negativ	4	30.8	10	45.5	
MP positiv	9	69.2	12	54.5	
**R-status dorsal**					*0.282*
R0CRM+	2	15.4	7	31.8	
R0CRM-	11	84.6	15	68.2	
**R-status medial**					*0.968*
R0CRM+	5	38.5	4	18.2	
R0CRM-	8	61.5	18	81.8	

CRM = circumferential resection margin; G = tumour grading; Ki-67 Index= Ki-67 proliferation index; L = lymphatic invasion; MP = mesopancreas; N = nodal stage; Pn = perineural invasion; T = tumour stage; V = venous invasion.

Supplemental Table S2: Correlation analysis of the 22 patients stratified according to resection type. Statistical significance was calculated using the chi-square test or Fisher's exact test, as appropriate. ** indicates a p-value ≤ 0.01; * indicates a p-value ≤ 0.05.

	**spleen-preserving distal pancreatectomy** n=12		**distal splenopancreatectomy** n=10		***p-value***
**Sex**	**n**	**%**	**n**	**%**	*0.145*
Male	6	50.0	8	80.0	
Female	6	50.0	2	20.0	
**T-stage**					*0.225*
T1 and T2	9	75.0	5	50.0	
T3 and T4	3	25.0	5	50.0	
**N-stage**					***0.003***
N0	11	91.7	3	30	
N1	1	8.3	7	70	
**Grading**					*0.495*
G1	5	41.7	3	30.0	
G2	7	58.4	6	60.0	
G3	0	0.0	1	10.0	
**Pn-status**					***0.041****
Pn0	12	100.0	7	70.0	
Pn1	0	0.0	3	3.0	
**V-status**					***0.015****
V0	12	100.0	6	60.0	
V1	0	0.0	4	4.0	
**L-Status**					***0.015****
L0	12	100.0	6	60.0	
L1	0	0.0	4	4.0	
**Ki-67-Index**					*0.184*
Ki-67 Index < 5%	7	58.3	3	30.0	
Ki-67 Index ≥ 5 %	5	41.7	7	70.0	
**MP-Status**					*0.639*
MP negativ	6	50.0	4	40.0	
MP positiv	6	50.0	6	60.0	
**R-status dorsal**					*0.867*
R0CRM+	4	33.3	3	30.0	
R0CRM-	8	66.7	7	70.0	
**R-status medial**					*0.840*
R0CRM+	2	16.7	2	20.0	
R0CRM-	10	83.3	8	80.0	

CRM = circumferential resection margin; G = tumour grading; Ki-67 Index= Ki-67 proliferation index; L = lymphatic invasion; MP = mesopancreas; N = nodal stage; Pn = perineural invasion; T = tumour stage; V = venous invasion.

Supplemental Table S3: Prediction analysis of the Mesopancreatic infiltration status by Ki-67 Index in PanNEN patients. Optimal cut-off point for Ki-67 Index was computed by ROC-analysis. Ordinal scaling of Ki-67 Index (<Cut-off and > Cut-off) was tested for distribution against MP infiltration status (Chi-Square test). Binary logistic regression analysis was used for prediction analysis

**Cut off by ROC Analysis**	5%

	MP Infiltration status		***p-value***
	MP not infiltrated	MP infiltrated	***0.001***
**Ki-67 Index < 5%**	11	5	***Sensitivity***
			76.19%
			Specificity
			78.57%
**Ki-67 Index ≥ 5 %**	3	16	***PPV***
			84.21%
			NPV
			68.75%

	p-value	Odds Ratio	95% CI
Binary logistic Regression analysis	***0.003***	11.73	2.31 – 59.54

CI = confidence interval; Ki-67 = Ki-67 proliferation index; MP = mesopancreas; NPV = negative predictive value; PPV = positive predictive value; ROC = receiver operating characteristic.

Supplemental Table S4: Prediction analysis of the Mesopancreatic infiltration status by tumorsize in PanNEN patients. Optimal cut-off point for tumorsize was computed by ROC-analysis. Ordinal scaling of tumorsize (<Cut-off and > Cut-off) was tested for distribution against MP infiltration status (Chi-Square test). Binary logistic regression analysis was used for prediction analysis

**Cut off by ROC Analysis**	21.5 mm

	MP Infiltration status		***p-value***
	MP not infiltrated	MP infiltrated	***0.001***
**Tumorsize < 21.5 mm**	11	7	***Sensitivity***
			66.67%
			Specificity
			78.57%
**Tumorsize ≥ 21.5 mm**	3	14	***PPV***
			82.35%
			NPV
			61.11%

	p-value	Odds Ratio	95% CI
**Binary logistic Regression analysis**	***0.013***	7.33	1.53 – 35.11

CI = confidence interval; MP = mesopancreas; NPV = negative predictive value; PPV = positive predictive value; ROC = receiver operating characteristic.

Supplemental Table S5: Univariate analysis of individual clinical and histopathological parameters regarding to overall survival

Univariate analysis	
	p-value
Median age (< vs. > median)	*0.544*
Sex (male vs. female)	*0.202*
Tumor location (head vs. tail)	*0.275*
T-stage (T1/T2 vs. T3/T4)	***0.027***
N-stage (N0 vs. N1)	*0.059*
Ki-67% (<5% vs. ≥5%)	*0.094*
Grading (G1 vs. G2/G3)	*0.322*
Pn (Pn0 vs. Pn1)	*0.401*
L (L0 vs. L1)	*0.385*
V (V0 vs. V1)	*0.511*
R-status (R0CRM- vs. R0CRM+)	*0.876*
MP-status (MP+ vs. MP-)	*0.138*

CRM = circumferential resection margin; G = tumour grading; Ki-67 = Ki-67 proliferation index; L = lymphatic invasion; MP = mesopancreas; N = nodal stage; Pn = perineural invasion; R = resection status; T = tumour stage; V = venous invasion.
